# Supplementary material for: Oligonucleotide Delivery across the Caco-2 Monolayer: The Design and Evaluation of Self-Emulsifying Drug Delivery Systems (SEDDS)
Source: Pharmaceutics. 2021 Mar 28;13(4):459. doi: 10.3390/pharmaceutics13040459 (PMC8066367; doi:10.3390/pharmaceutics13040459)
Supplement: Supplementary file 1 [file pharmaceutics-13-00459-s001.pdf]

pharmaceutics

Supplementary Materials: Oligonucleotide Delivery across the Caco-2 Monolayer: The Design and Evaluation of Self-Emulsifying Drug Delivery Systems (SEDDS)

Jana Kubackova, Ondrej Holas, Jarmila Zbytovska, Barbora Vranikova, Guanghong Zeng, Petr Pavek and Anette Mullertz

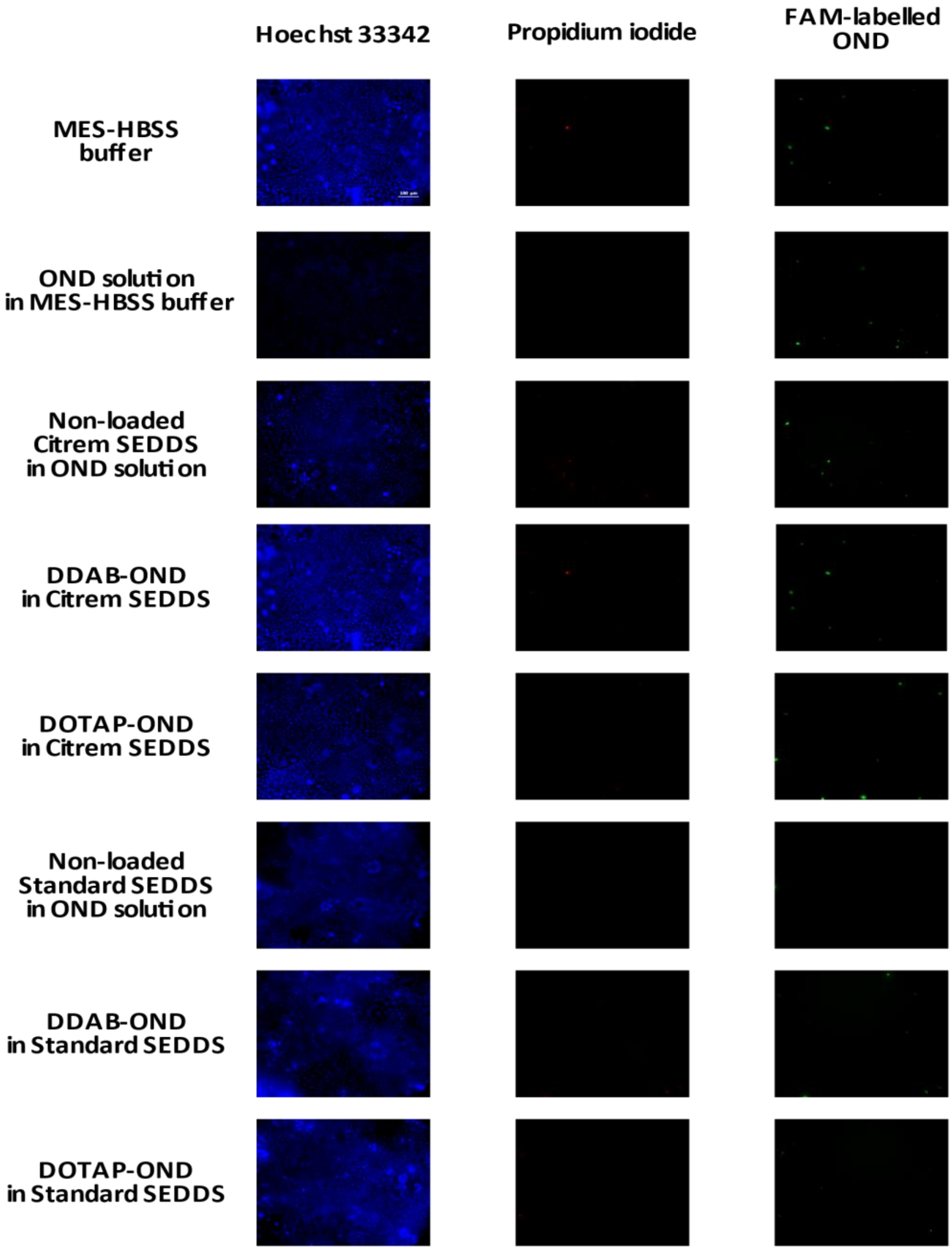

Figure S1. Fluorescence microscope images. Images show blue Hoechst 33342 staining of cell nuclei, red propidium iodide dye showing dead cells and green FAM-labeled OND. The scale in the first image represents 100 µm and is applicable to all images. OND-oligonuclotide, SEDDS -self-emulsifying drug delivery system.
